# Refractory Cancer Pain in a Pediatric Patient With Advanced Malignant Peripheral Nerve Sheath Tumor: A Total Pain-Oriented Palliative Care Approach

**DOI:** 10.7759/cureus.103114

**Published:** 2026-02-06

**Authors:** Wania Imtiaz, Iqra Muneeb, Irum Ghafoor, Haroon Hafeez

**Affiliations:** 1 Internal/Palliative Medicine, Shaukat Khanum Memorial Cancer Hospital and Research Centre, Lahore, PAK

**Keywords:** multimodal analgesia, pediatric palliative care, quality of life, refractory cancer pain, total pain

## Abstract

Malignant peripheral nerve sheath tumors (MPNSTs) are a rare and aggressive form of pediatric sarcomas, which are often accompanied by uncontrolled, intense pain, especially during advanced cancer stages. Pediatric oncology pain is often multidimensional and entails physical, psychological, and social facets of distress. Here, we describe the case of an eight-year-old girl with metastatic MPNST with refractory mixed nociceptive and neuropathic pain, even with increased opioid therapy, add-on analgesics, palliative radiotherapy, and peripheral nerve blocks. The detailed palliative care plan, based on the total pain framework, covered not only the physical symptoms but also psychological distress, as well as family-related issues. Multimodal interventions in the form of constant subcutaneous infusions, psychiatric, and tunneled epidural analgesia ultimately managed the symptoms. This case highlights the importance of identifying and dealing with total pain in pediatric oncology. Interventional analgesic methods could be useful, but they are most useful when combined within an overall palliative care model that is based on comfort, dignity, and quality of life.

## Introduction

Pain is one of the most stressful manifestations in children with advanced cancer and is often under-evaluated and under-managed [1]. Pain in pediatric oncology is hardly physical as it is often coupled with psychological distress, anxiety, family pain, and existential anxiety to increase the total symptom burden. The connection between the physical, psychological, social, and spiritual aspects of suffering is highlighted in the concept of total pain by Dame Cicely Saunders; this is an effective model to use in palliative care in the context of life-limiting illness [2].

Malignant peripheral nerve sheath tumors (MPNSTs) are non-epithelial, rare, soft-tissue sarcomas with an aggressive course and prognosis in the metastatic stage [3]. Their neural invasion and skeletal predilections make the affected children susceptible to severe and essentially refractory pain. Although the primary approach to cancer pain management is opioids and adjuvant drugs, some patients may require other modalities. In this report, a case of a total pain approach is reported as a palliative intervention in a child diagnosed with advanced MPNST.

## Case presentation

**Figure 1:**
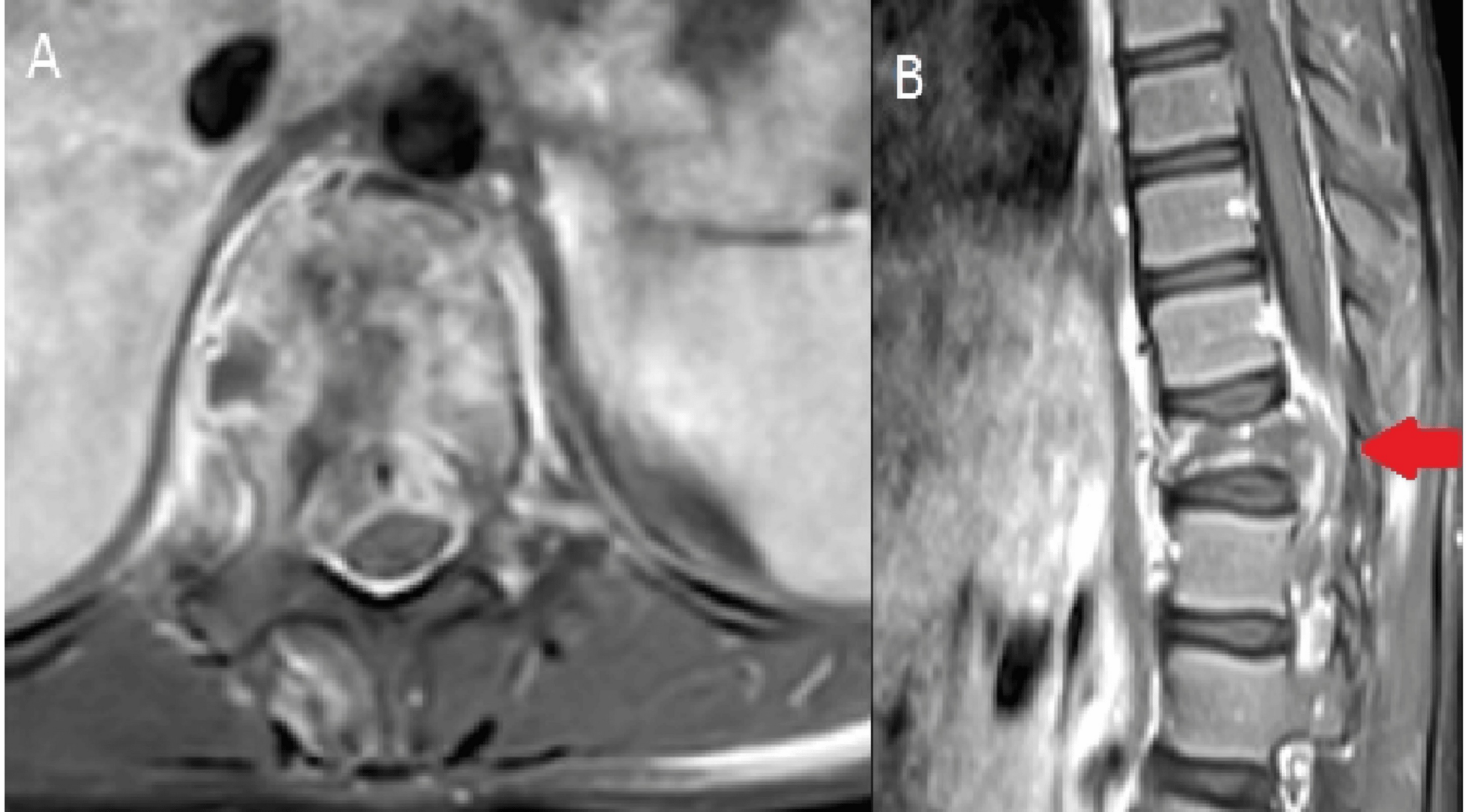
MRI of the thoracic spine showing a T11 vertebral body pathological fracture with an enhancing extraosseous soft-tissue component, causing effacement of the thecal sleeves and cord compression. (A) Axial view. (B) Sagittal view.

**Figure 2:**
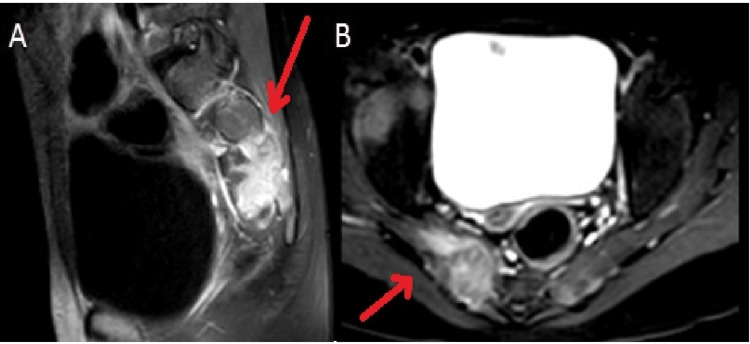
MRI of the lumbar spine showing an osseous metastatic deposit involving the right sacral ala with an associated enhancing extraosseous soft-tissue mass extending into the adjacent sacral neural foramena and causing nerve root compression. (A) Sagittal view. (B) Axial view.

An eight-year-old Afghan girl with a known MPNST with an origin in the left axillary and retropectoral area was referred with progressive metastatic disease even after oncologic therapy. Imaging classified the skeletal metastases as extensive, with a pathological fracture of the T11 spine vertebra and spinal cord compression, along with the involvement of the pelvic and sacrococcygeal area (Figures 1, 2).

She had undergone prior palliative radiotherapy to symptomatic areas with temporary resultant pain relief. As the disease progressed, she was referred to Palliative Medicine and was admitted due to a severe pain crisis, which was characterized by constant mixed nociceptive and neuropathic pain. The Numerical Pain Scale and Wong-Baker FACES Pain Rating Scale were used for pain assessment. The crisis was also characterized by high levels of anxiety, agitation, and sleep disturbance, resulting in a psychological element to her misery that was significant.

Early management included an escalation of oral and subcutaneous morphine with combined adjuvant agents, which included gabapentin, amitriptyline, carbamazepine, non-steroidal anti-inflammatory drugs, and topical ketoprofen. Optimization of pharmacologic therapy was still insufficient to control pain.

Several sessions with the patient, conducted by the psychology team, revealed that she was stressed about her long separation from her mother and sisters and that she had not seen them in the last year after beginning her cancer treatment. Therefore, the family was contacted in Afghanistan to support her. A psychiatric consultation was sought due to persistent agitation and emotional discomfort, and haloperidol and antidepressant therapy were introduced.

Focal limb peripheral nerve blocks with phenol (right saphenous nerve at the lower third of the thigh, genicular nerves of the right knee, and right sciatic nerve at the popliteal level) were done inpatient due to ineffective analgesia and resulted in short-term effects.

**Figure 3:**
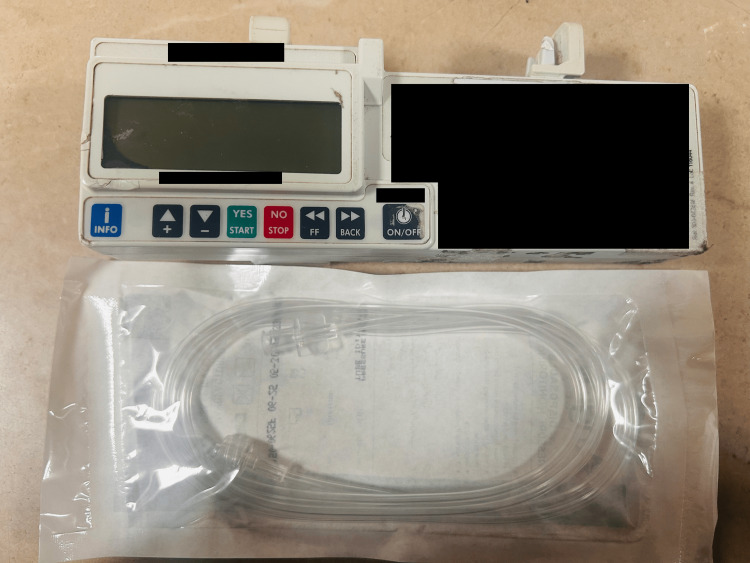
Portable infusion pump used for the continuous infusion of analgesia for pain management.

The patient’s medical history was associated with severe grade 4 oral mucositis and oral candidiasis, which restricted oral intake and added to the condition of physical discomfort. Given the refractory pain and the progressive condition accompanied by decreased oral intake, a tunneled epidural catheter was inserted at the L3-L4 level, which administered a continuous local anesthetic, bupivacaine, together with a subcutaneous morphine and midazolam infusion as the patient remained dependent on parenteral analgesia due to oral candidiasis (Figure 3).

This intervention led to significant improvement in comfort, pain behavior reduction, and increased tolerance to routine care (Table 1).

**Table 1:** Pain scores before and after interventions.

Interventions	Pain score
No analgesia	10
Oral analgesia (non-opioids)	10
Oral analgesia (opioids + adjuncts)	9
Postoperative day after tunneled epidural catheter placement	
1	7
3	5
7	5
Postoperative day after tunneled epidural catheter placement and addition of continuous subcutaneous infusion of morphine and midazolam	
10	3
14	2
16	0

The Lansky Play-Performance Scale was used to assess performance status, but it continued to deteriorate over the course of admission. The family was engaged in goal-of-care conversations, and a do-not-resuscitate order was determined. Further treatment was directed at only comfort-based palliation. The patient passed away comfortably in the hospital among her family.

## Discussion

This case study shows that pain can be quite complex and multifaceted in children with advanced malignant conditions and that a total pain model is vital in the context of pain management. Although the issue of physical pain was central, psychological pain, suffering due to treatment, and family anxiety played a significant role in the overall patient experience.

Total pain in pediatric palliative care

Pain in patients with advanced malignancy is a multidimensional experience often beyond the confines of nociceptive or neuropathic processes. The notion of total pain, first introduced by Cicely Saunders, highlights the fact that pain is not only determined by physical pathology but also by psychological, social, and spiritual distress [2]. These domains rarely do not interact, which increases the burden of symptoms and makes the pain refractory to exclusively pharmacologic or interventional strategies [4].

In children and young adults with progressive cancer, the intensity of total pain can be especially high due to vulnerability (developmental), dependence (causation), disruption of schooling, and existential distress of both the patient and the family [5]. Therefore, identification of total pain is critical in the practice of palliative care as a multidisciplinary, holistic approach to symptom management is often required, beyond analgesic escalation [6].

This case report presents how the total pain framework can be used to manage refractory cancer-related pain and explains how combination palliative interventions that consider the physical, psychological, social, and spiritual aspects of patients can significantly enhance comfort and quality of life.

In addition to the physical pain imposed by progressive malignant disease, other important contributors to the distress included non-physical factors in this patient. Psychological pain was in the form of anxiety, fear, and emotional distress because of the progression of the disease and the inability to perform normal childhood activities. Caregiver exhaustion, family role disruption, and extended hospitalization were the sources of social pain. Spiritual and existential distress was manifested in withdrawal, decreased engagement, and abstracted fear about suffering and uncertainty.

A systematic total pain evaluation provided the care team with the means of identifying these domains of overlap and guided a far-reaching, interdisciplinary control plan. The presence of psychiatric support and family-focused communication as the means of preventing psychological and emotional misery turned out to be important in achieving comfort in the case.

Role of palliative radiotherapy

Radiotherapy has been identified as a fundamental modality in the management of oncologic pain, especially in conditions characterized by the presence of metastases to the bone and the spinal region [7]. For pediatric patients, palliative radiotherapy can decrease tumor burden and pain and minimize neurologic dysfunction. However, the analgesic response tends to have temporal latitude and may be temporary, particularly with the quickly developing disease. Radiotherapy in the current case only provided a modest benefit, which highlights its importance as a component in an overall approach to pain management, as opposed to a stand-alone intervention.

Role of peripheral nerve blocks

Peripheral nerve blocks are a more precise solution to pain localized to particular neural areas and could decrease overall opioid use [8]. This case illustrated short-term symptomatic effects of selective nerve blockages but was limited by the widespread and progressive nature of metastatic pain. Nerve blocks are crucial as adjunctive or bridging therapies, but often cannot help when it comes to disseminated malignancy.

Role of epidural analgesia

Neuraxial analgesia is one of the stronger alternatives to refractory cancer pain when other methods fail. Tunneled epidural catheters provide a method of continuous regional analgesia and may significantly reduce the adverse effects of opioids [9]. In this patient, an epidural local anesthetic infusion provided significant symptom alleviation and improved the quality of life in the terminal days of the disease.

Ethical and family-centered care

Palliative care involvement early on enhanced shared decision-making and mutually consistent interventions with the ultimate goal of comfort. Avoidance of non-beneficial treatment and giving precedence to the end-of-life dignity are key ethical concerns in pediatric cancer care [10].

## Conclusions

Pediatric patients with advanced malignancy and the oncologic pain experienced in the refractory stage are often multifactorial and require a holistic approach. This case shows how the implementation of the concept of total pain helps clinicians treat physical symptoms and respond to the distress experienced by their patients while addressing psychological and family distress. Interventional pain management approaches can bring much relief, although these methods must be incorporated as part of an all-inclusive, palliative care plan to make the end of life more comfortable, dignified, and with improved quality of life.
